# Optimization of a protocol for contrast-enhanced four-dimensional computed tomography imaging of thoracic tumors using minimal contrast agent

**DOI:** 10.1007/s00066-021-01836-8

**Published:** 2021-09-02

**Authors:** Hongya Dai, Dingqiang Yang, Lu Chen, Yibing Zhou, Xiaojing Wen, Jianguo Sun, Guanghui Li

**Affiliations:** Department of Oncology, Xinqiao Hospital, Army Medical University, Chongqing, China

**Keywords:** 4D computed tomography, Contrast agent, Radiotherapy, CT image enhancement, Thoracic tumors

## Abstract

**Purpose:**

The accuracy of target delineation for node-positive thoracic tumors is dependent on both four-dimensional computed tomography (4D-CT) and contrast-enhanced three-dimensional (3D)-CT images; these scans enable the motion visualization of tumors and delineate the nodal areas. Combining the two techniques would be more effective; however, currently, there is no standard protocol for the contrast media injection parameters for contrast-enhanced 4D-CT (CE-4D-CT) scans because of its long scan durations and complexity. Thus, we aimed to perform quantitative and qualitative assessments of the image quality of single contrast-enhanced 4D-CT scans to simplify this process and improve the accuracy of target delineation in order to replace the standard clinical modality involved in administering radiotherapy for thoracic tumors.

**Methods:**

Ninety consecutive patients with thoracic tumors were randomly and parallelly assigned to one of nine subgroups subjected to CE-4D-CT scans with the administration of contrast agent volume equal to the patient’s weight but different flow rate and scan delay time (protocol A1: flow rate of 2.0 ml/s, delay time of 15 s; A2: 2.0 ml/s, 20 s; A3: 2.0 ml/s, 25 s; B1: 2.5 ml/s, 15 s; B2: 2.5 ml/s, 20 s; B3: 2.5 ml/s, 25 s; C1: 3.0 ml/s, 15 s; C2: 3.0 ml/s, 20 s; C3: 3.0 ml/s, 25 s). The Hounsfield unit (HU) values of the thoracic aorta, pulmonary artery stem, pulmonary veins, carotid artery, and jugular vein were acquired for each protocol. Both quantitative and qualitative image analysis and delineation acceptability were assessed.

**Results:**

The results revealed significant differences among the nine protocols. Enhancement of the vascular structures in mediastinal and perihilar regions was more effective with protocol A1 or A2; however, when interested in the region of superior mediastinum and supraclavicular fossa, protocol C2 or C3 is recommended.

**Conclusion:**

Qualitatively acceptable enhancement on contrast-enhanced 4D-CT images of thoracic tumors can be obtained by varying the flow rate and delay time when minimal contrast agent is used.

## Introduction

Respiration motion can cause significant volumetric deformation of tumor images in conventional three-dimensional computed tomography (3DCT), which leads to inaccurate target volume delineation and may possibly impact the following treatment course and outcome. To explicitly include organ/target motion in treatment planning and delivery, respiration-correlated four-dimensional computed tomography (4D-CT) is needed, which has the ability to record tumor motion and can be used to obtain respiration artifact-free CT images of thorax tumors, thereby allowing tumor motion assessments for individual patients [1–8]. In most radiotherapy departments, 4D-CT scanning has become the mainstream technology for CT simulation in patients with thorax tumors.

The 4D-CT technology, however, has introduced new challenges into clinical practice. In administering radiotherapy to treat thoracic tumors that involve mediastinal and/or cervical lymph nodes, challenges with regard to tumor visualization still persist with the use of 4D-CT technology alone in the absence of contrast enhancement. The advantage of contrast-enhanced CT images in terms of identifying lesion volume, lymph nodes, and surrounding invasion has been recognized [9, 10]. McGibney et al. reported that intravenous contrast-enhanced CT is widely utilized to improve the delineation of the tumor volume and lymph node areas in thoracic radiotherapy, and can reduce the volume of gross target volumes (GTVs) by 22–34% [10]. In order to combine the advantages of respiratory management and enhancement, some departments of radiotherapy perform image registration on 4D-CT images and on contrast-enhanced (breath-hold) 3D-CT images [11, 12]. Under these circumstances, however, this technique is still not the most effective recommendation because 3D-CT images that do not account for tumor motion are prone to artifacts, and the deviation of 4D and 3D images is large. On considering this, it is natural to think of combining both modalities into one technique in order to overcome the aforementioned limitations. The integration of contrast enhancement into 4D-CT imaging is a desirable goal for accurate tumor delineation.

The most significant difference between 4D-CT and 3D-CT scanning is the acquisition time. Although a normal helical scan can be acquired in a few seconds, a 4D-CT scan may take up to 1 min or more for the same length. It has been deemed that it is not possible to maintain contrast enhancement through the entire thoracic 4D-CT duration. Therefore, manufacturers have not provided a scanning protocol for contrast-enhanced 4D-CT imaging. However, there are still other studies in the literature that suggest that the application of contrast-enhanced 4D-CT imaging scans by administering a synchronized intravenous contrast injection improves target delineation in liver cancer, thoracic esophageal cancer, and pancreatic tumors, thereby, making this a feasible technique [13–15]. For example, the administration of a 150 ml contrast at a flow rate of 5 ml/s during a 4D-CT scan in order to improve the delineation of liver tumors has been reported [15]. Furthermore, patients have been administered a total contrast medium volume of 140 ml at a rate of 2.9 ml/s during a contrast-enhanced 4D-CT scan for target volume definition in pancreatic ductal adenocarcinoma [13]. These protocols were specifically designed based on the experience of teams comprised of physicians and physicists in different radiotherapy departments. However, in our department, a wide scan range including neck and entire chest is required for thoracic tumors, and there is no universal agreement in the literature regarding the parameters that are appropriate for the optimization of 4D-CT imaging of thoracic tumors.

Thus, we attempted to develop suitable injection parameters for 4D-CT scanning with a volume of contrast agent equivalent to that used in contrast-enhanced 3D-CT scans to improve delineation of thoracic tumors for radiotherapy. Insufficient opacification of vessels is the most frequent reason for failure in accurate delineation; thus, increased enhancement of vessels also means enhancement of the adjacent target and lymph nodes, which makes it easier to distinguish the boundaries between them. This study consisted of two steps. The first was to quantitatively and qualitatively analyze the enhancement of vessels to determine the optimal 4D-CT scanning acquisition protocol for thoracic tumors. The second was to verify the visibility of target volume (primary tumor and nodal areas, especially with invasion of the mediastinum) when the recommended protocol for contrast-enhanced 4D-CT is used.

## Materials and methods

### Patients

From July 2017 to February 2019, 90 patients (70 men and 20 women; age range 24–75 years, median age 46 years) with thoracic tumors were randomly and parallelly assigned to nine groups for CE-4D-CT scanning. This trial was verified by the Chinese Clinical Trial Registry (registration number: ChiCTR-DDD-16009645; http://www.chictr.org.cn/index.aspx) and was approved by the institutional ethics review board (ethical number: 2017-019-01). The inclusion criteria were an Eastern Cooperative Oncology Group (EGOG) performance status of 0 or 1, and adequate hepatic, renal, and hematologic function. Owing to the risks of negative reactions to the contrast agent, the exclusion criteria were allergic disorders, clinically significant cardiovascular disease, clinically significant ventilation function disability, and medically uncontrolled hypertension. Written informed consent for study participation was obtained from all patients prior to the study. All the procedures were performed in accordance with the 1964 Helsinki declaration.

### Scanning protocols and contrast agent injection

This study was performed using a commercial CT system (16-detector, Philips Healthcare, Best, The Netherlands) that involved the use of pulmonary gating to ensure high-quality imaging scans of respiratory motion. Free-breathing scanning could be performed with the use of a respiratory bellows belt that is a deformable rubber belt placed across the patient’s waist and aids in measuring changes in lung volume; this technique was able to generate a breathing signal corresponding to the lung volume. The patients were scanned in the supine position within a personalized body-fix thermoplastic mask specifically modeled based on the body shape to ensure that the same position was maintained during imaging and radiotherapy (RT) sessions.

The acquisition parameters of contrast-enhanced 4D-CT (CE-4D-CT) scan were as follows: voltage, 120 kV; 400 mAs/slice; table speed, 7.2 mm/s; 5 mm slice thickness; collimation, 16 × 1.5 mm; pitch, 0.15; scanning direction, craniocaudal; 0.5 s rotation time. The 4D-CT images were binned into 10 respiratory phases, and all the phases were imported to Eclipse (v8.6, Varian Medical Systems, Palo Alto, CA, USA). The scanning procedure consisted of a scout view and contrast-enhanced 4D-CT scan. The scout view was performed to set the craniocaudal limits of the scan, and the scan covered the region from the throat to diaphragmatic dome in all the patients in this study.

Iodixanol (320 mg of iodine/ml; Jiangsu Hengrui Pharmaceutical Co. Ltd., Lianyugang, China) was used as the contrast agent and was administered using a high power injector (MedradVistron CT^TM^ injection system, Indianola, PA, USA) through a plastic intravenous catheter typically placed in an antecubital vein. Contrast enhancement at CT is determined by numerous interacting factors [16–19] such as technique-related factors and patient-related factors. The key patient-related factors affecting contrast enhancement are patient body size and cardiac output; the most important technique-related factors include contrast material volume and delay time, and rate of injection. Easy to operate and standardize, we just take into account following factors: flow rate, volume, and scan delay time. Patients were randomly selected to undergo one of the following nine CE-4D-CT subgroups: protocols A1—flow rate of 2.0 ml/s, a scan delay of 15 s; protocols A2—flow rate of 2.0 ml/s, a scan delay of 20 s; protocols A3—flow rate of 2.0 ml/s, a scan delay of 25 s; protocols B1—flow rate of 2.5 ml/s, a scan delay of 15 s; protocols B2—flow rate of 2.5 ml/s, a scan delay of 20 s; protocols B3—flow rate of 2.5 ml/s, a scan delay of 25 s; protocols C1—flow rate of 3.0 ml/s, a scan delay of 15 s; protocols C2—flow rate of 20 s; protocols C3—flow rate of 3.0 ml/s, a scan delay of 25 s. The volume of contrast agent in all CE-4D-CT scans was 1:1 linear weight of the patient that was used in routine contrast-enhanced 3D-CT in our department.

### Quantitative image analysis

The quantitative assessment of the contrast-enhanced 4D-CT images was performed by a radiation oncologist with more than 5 years of experience in target delineation. Insufficient opacification of vessels is the most frequent reason for failure to achieve accurate delineation; therefore, we mainly measured the CT values (Hounsfield unit, HU) of vessels. For each scan, circular regions of interest (ROI) were drawn on definitive axial slices to determine the mean HU values and standard deviations (SD) of the following structures: the thoracic aorta, pulmonary artery stem, pulmonary veins, carotid artery, and jugular vein. In addition, 50% of the CT image series phase (expiration phase) was selected in this study. The quantitative degrees of contrast enhancement were expressed as CT values (HU) on contrast-enhanced axial images.

### Qualitative image analysis

Two experienced radiation oncologist who were blinded to patient clinical information and the CT imaging parameters retrospectively and independently reviewed the 4D-CT images in the mediastinum window (window width of 360 HU and window level of 60 HU). They qualitatively assessed all the images in terms of the following parameters: (1) regional vessel definition, mainly involving the detail and enhancement and (2) the enhancement of tumors and lymph nodes close to vessels along with the ability to discriminate them from the surrounding normal tissues. A total of 5 grades were assigned based on the radiation oncologists’ subjective experience in conventional clinical acquisitions, as follows: 1 = “poor”, 2 = “unsatisfactory”, 3 = “sufficient”, 4 = “good” and 5 = “excellent” [11]. Grade 1 was assigned when image quality was quite poor and no enhancement was observed, grade 3 was assigned when images were degraded within the acceptable limit that did not hamper the interpretation, grade 5 was assigned when the enhancement of vascular segments and adjacent lymph nodes were clearly visualized, and grades 2 and 4 were defined as being intermediates between grades 1 and 2 and grades 3 and 5, respectively [20].

### Cases and target volume visibility

Because the primary purpose of CE-CT images is to identify lesion volume, lymph nodes, and surrounding invasion, it is necessary to analyze the visibility of the target volume (primary tumor, lymph node region, and mediastinal invasion). We randomly selected four cases with disease confirmed by diagnosis to analyze their target volume visibility. In the same position, plain 3D-CT scans were performed first, followed by CE-4D-CT scans, and the injection parameters of CE-4D-CT were selected based on the recommendation of this paper. We suggest that the visibility of the target volume is not merely that it exhibits enhancement, but that it is sufficient to recognize the boundary between it and the surrounding structure (such as low enhancement or no enhancement in the target volume, but there is enhancement in the adjacent vessels). If the boundary of the target region were recognized, we assume that the visibility of the target volume is positive; otherwise, it is negative.

### Statistical analysis

Statistical analyses were performed using the SPSS version 17.0 software (IBM, Chicago, IL, USA). A one-way analysis of variance (ANOVA) and post hoc Tukey test were performed to evaluate the differences in patient age and weight among the nine groups. The inequality of variances in the HU values and score among the nine protocols was tested with multiple comparisons in the ANOVA (Tukey). Pair-wise comparisons were performed to evaluate the differences in qualitative ratings among the nine protocols.

## Results

Patient demographics and the parameters used in the contrast enhanced-4D-CT protocols are outlined in Table 1.

**Table 1:** Patient characteristic, flow rate of contrast material and scan delay for each protocol

Protocol	No. of patients	Age (years)	Body weight (kg)	Flow rate (ml/s)	Scan delay (s)
A1	10	60.8 ± 9.1	63.6 ± 11.1	2.0	15
A2	10	49.8 ± 10.1	67.6 ± 7.1	2.0	20
A3	10	59.8 ± 8.7	62.6 ± 6.4	2.0	25
B1	10	54.7 ± 13.2	62.2 ± 12.5	2.5	15
B2	10	55.6 ± 10.3	65.4 ± 11.6	2.5	20
B3	10	52.9 ± 8.1	65.4 ± 13.0	2.5	25
C1	10	57.0 ± 8.6	64.7 ± 9.3	3.0	15
C2	10	58.8 ± 6.8	59.1 ± 5.8	3.0	20
C3	10	51.3 ± 13.2	59.5 ± 9.5	3.0	25

Flow rate of contrast material and scan delay for protocol A1–C3. Data of age and weight were mean ± standard deviation. No significant different was found in age (*P* = 0.280) and weight (*P* = 0.635) between the 9 groups

## Quantitative image analysis

Regarding the enhancement of vascular structures, the mean and standard deviation (SD) of HU are provided in Table 2.

**Table 2:** The average Hounsfield unit (HU) values of vessels in all protocols (mean ± standard deviation)

Protocol	Thoracic aorta	Pulmonary artery stem	Pulmonary veins	Carotid artery	Jugular vein
A1	223 ± 34^a^	188 ± 33^a^	157 ± 28^a^	184 ± 45^a^	84 ± 46^a^
A2	235 ± 26^a^	183 ± 33^b^	161 ± 29^b^	229 ± 36	155 ± 66
A3	203 ± 54	148 ± 19	131 ± 13	221 ± 60	161 ± 52
B1	216 ± 40	152 ± 38	129 ± 21	219 ± 43	107 ± 49
B2	204 ± 37	148 ± 21	140 ± 24	256 ± 27	146 ± 36
B3	192 ± 52	142 ± 19	128 ± 26	247 ± 36	173 ± 59
C1	209 ± 49	139 ± 12	125 ± 18	242 ± 60	114 ± 50
C2	189 ± 43	144 ± 21	129 ± 14	270 ± 58^b^	186 ± 79^b^
C3	145 ± 19^b^	124 ± 13^c^	107 ± 16^c^	218 ± 47	225 ± 65^c^

Thoracic aorta — ^a^Difference between protocols A1 and A2 and protocols C2 and C3 were statistically significant (*P* < 0.05). ^b^Protocol C3 differed significantly from other protocols (*P* < 0.05)

Pulmonary artery stem — ^a^Protocol A1 differed significantly from other protocols except A2 (*P* < 0.05). ^b^Protocol A2 differed significantly from other protocols except protocol A1 and B1 (*P* < 0.05). ^c^Protocol C3 differed significantly from other protocols except B1 (*P* < 0.05)

Pulmonary veins — ^a^Difference between protocol A1 and protocols A3, B1, B3, C1, C2 and C3 were statistically significant (*P* < 0.05). ^b^Protocol A2 differed significantly from other protocols except A1 and B2 (*P* < 0.05). ^c^Protocol C3 differed significantly from other protocols except B1 (*P* < 0.05)

Carotid artery — ^a^Protocol A1 differed significantly from other protocols (*P* < 0.01). ^b^Difference between protocol A3 and C2 was statistically significant (*P* < 0.05)

Jugular vein — ^a^Protocol A1 differed significantly from other protocols except B2 and C1 (*P* < 0.05). ^b^Difference between protocol C2 and protocols A1, B1 and C1 were statistically significant (*P* < 0.05). ^c^Difference between protocol C3 and protocols A1, B1, B2 and C1 were statistically significant (*P* < 0.05)

The two largest HUs of thoracic aorta were 223 ± 34 and 235 ± 26 in the groups involving protocol A1 and A2, respectively; the average HUs were observed to be greater in protocols A1 and A2 than those in protocols C2 and C3 (*P* < 0.05). The smallest HUs of the thoracic aorta was 145 ± 19 in group C3, which were significantly smaller than those for the other protocol groups (*P* < 0.05).

The two largest HUs of the pulmonary artery stem were 188 ± 33 and 183 ± 33 in the groups involving protocol A1 and A2 respectively; the average HUs was greater in group A1 than in the other groups except A2 (*P* < 0.05), and those values were greater in the group of protocol A2 than in the other protocols except A1 and B1 (*P* < 0.05); the smallest HUs of the pulmonary artery stem was 107 ± 16 in group C3, and those differed significantly from other protocols except B1 (*P* < 0.05).

Similar to the results of the thoracic aorta and the pulmonary artery, the two largest HUs of pulmonary veins were 157 ± 28 and 161 ± 29 in the groups involving protocols A1 and A2, respectively; the HUs of the pulmonary veins were greater in groups A1 and A2 than those in protocols A3, B1, B3, C1, C2 and C3 (*P* < 0.05); protocol C3 was significantly smaller than other protocols except B1 (*P* < 0.05).

The HU value of the carotid artery was 184 ± 45 in protocol A1 and significantly smaller than the other protocols (*P* < 0.05); the largest HUs was 270 ± 58 in protocol C2.

The smallest HU value of the jugular vein was 84 ± 46 in protocol A1; the two largest values were 186 ± 79 and 225 ± 65 in the groups involving protocol C2 and C3 respectively.

## Qualitative image analysis

The quality of the depiction of the thoracic aorta, pulmonary artery stem, and pulmonary veins were higher with protocols A1 and A2, while the depiction of the carotid artery and jugular veins were higher with protocols C2 and C3 (Table 3).

**Table 3:** Results of the qualitative analysis performed on the contrast enhanced-4D-CT images (enhancement range: 1–5)

Protocol	Thoracic aorta	Pulmonary artery stem	Pulmonary veins	Carotid artery	Jugular veins
A1	4.8 ± 0.4^a^	3.9 ± 1.0^a^	3.4 ± 0.7^a^	4.0 ± 1.0	1.5 ± 1.0
A2	5.0^b^	4.1 ± 0.9^a^	3.5 ± 0.5^a^	4.7 ± 0.5	2.7 ± 1.4
A3	3.9 ± 1.3	2.2 ± 0.6	2.0	4.6 ± 1.3	3.4 ± 1.2
B1	4.4 ± 1.1	2.6 ± 1.1	2.1 ± 0.3	4.4 ± 1.1	1.8 ± 1.0
B2	3.8 ± 1.3	2.4 ± 0.7	2.9 ± 1.0	4.3 ± 0.8	2.5 ± 1.1
B3	3.4 ± 1.4	2.2 ± 0.6	2.1 ± 0.6	4.8 ± 0.6	3.1 ± 1.5
C1	4.1 ± 1.3	2.3 ± 0.5	2.0	4.4 ± 1.3	2.0 ± 1.1
C2	3.6 ± 1.2	2.6 ± 0.7	2.2 ± 0.4	4.9 ± 0.3^a^	4.1 ± 0.9^a^
C3	2.2 ± 0.4	2.5 ± 0.5	1.7 ± 0.5	4.5 ± 0.7	4.5 ± 0.8^a^

Thoracic aorta — values indicated with ^a^were significantly higher than protocols C2 and C3; values indicated with ^b^were significantly higher than protocols A3, C2 and C3

Pulmonary artery stem — values indicated with ^a^were significantly higher with protocols A1 and A2

Pulmonary veins — values indicated with ^a^were significantly higher than other groups except B2

Carotid artery — values indicated with ^a^was significantly higher with protocol C2 compared to with protocol A1

Jugular vein — values indicated with ^a^were significantly higher with protocols C2 and C2 compared to other protocols

The enhanced values (HUs and grade) of vessels located in the mediastinum (thoracic aorta, pulmonary artery stem and pulmonary veins) were larger in groups of A1 (flow rate is 2.0 ml/s; scan delay is 15 s) or A2 (flow rate is 2.0 ml/s; scan delay is 20 s); however, the enhanced values of vessels in the neck including carotid artery and jugular vein were smallest in group A1. In group C2, the enhanced values of the carotid artery and jugular vein were the largest; however, the enhanced values of the thoracic aorta, pulmonary artery stem and pulmonary veins were insufficient in this protocol. The CT slice images of each structure in protocols A1–C3 are provided in Fig. 1.

**Fig. 1:**
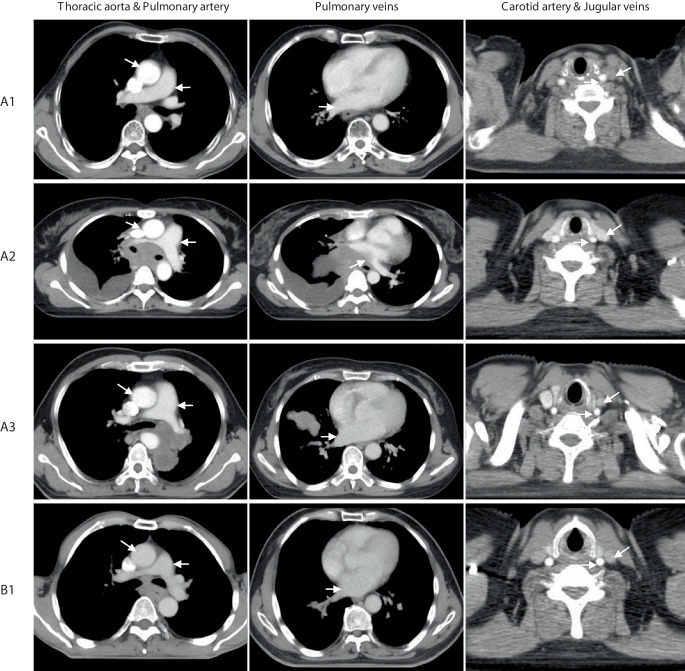
Contrast enhancement of vessels in protocols A1–C3 (as indicated by the *white arrow*)

**Fig. 1:**
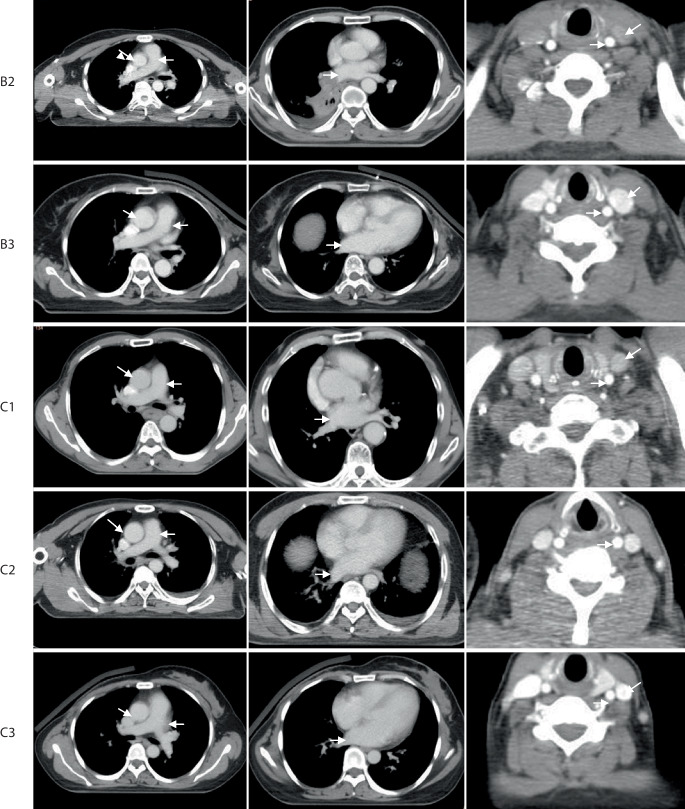
continued

## Case presentations

### Case 1

**Fig. 2:**
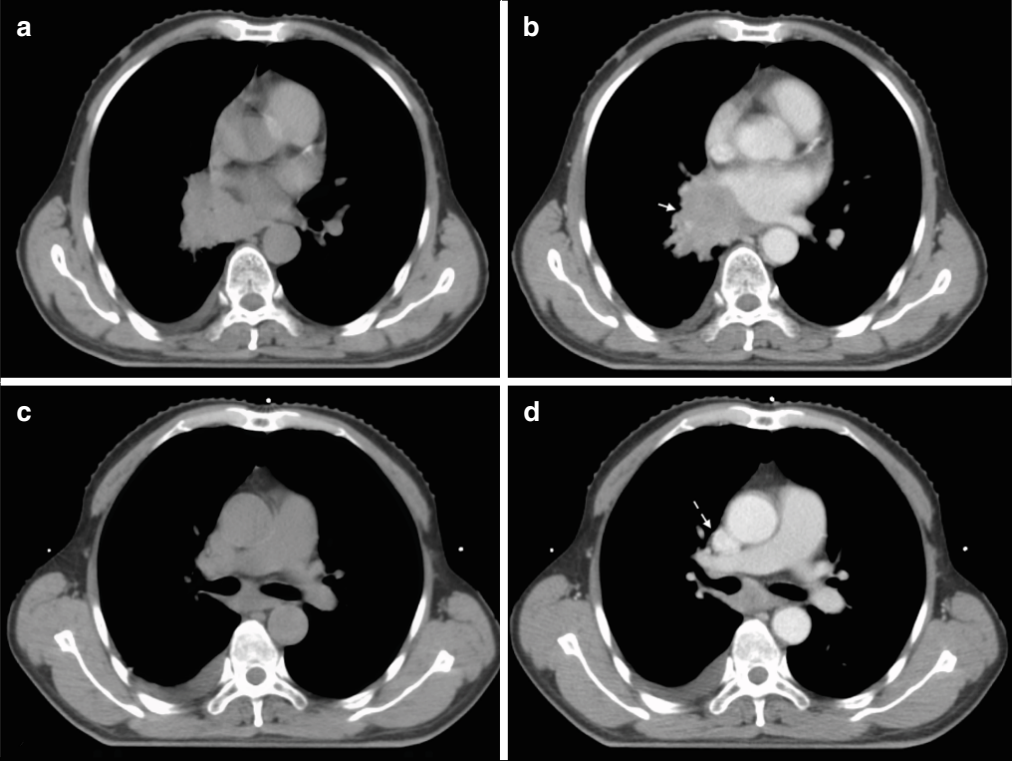
The Hounsfield units (HUs) of primary lesion in plain 3D-CT (**a**) and CE-4D-CT (**b**) were 56 ± 7 and 71 ± 10, respectively; the HUs of mediastinal lymph nodes in plain 3D-CT (**c**) and CE-4D-CT (**d**) were 39 ± 6 and 55 ± 15, respectively. The lesion in **a** and the lymph node in **c** are ill-defined, but well-defined in enhanced **b** (*solid arrow*) and **d** (*dotted arrow*)

Endoscopy showed right-located primary squamous cell lung cancer, and hilum and mediastinal lymph node metastasis in the PET-CT. The CE-4D-CT scan was run using protocol A1 (flow rate of 2.0 ml/s; a scan delay of 15 s). Although the enhancement of lymph nodes and primary lesion were modest compared with vascular enhancement, it could be clearly distinguished from adjacent vessels and normal tissue with visibility being classified as “positive” (Fig. 2).

### Case 2

**Fig. 3:**
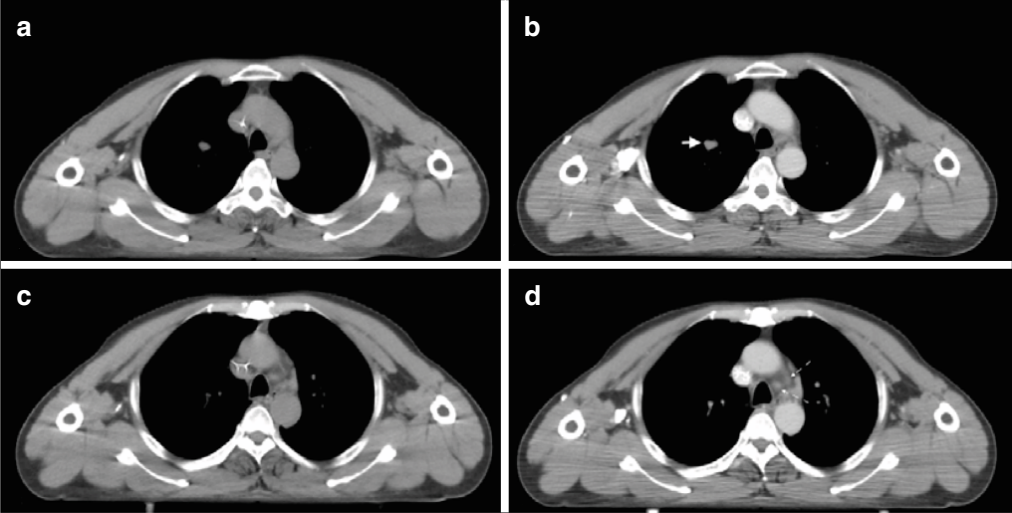
The Hounsfield units (HUs) of lesion in plain 3D-CT (**a**) and CE-4D-CT (**b**) were 13 ± 3 and 42 ± 7, respectively; and the HUs of mediastinal lymph nodes in plain 3DCT (**c**) and CE-4D-CT (**d**) were 1 ± 25 and 17 ± 26, respectively. The nodes were clearly identified (*dotted arrow*)

Right upper lobe small cell lung cancer. The CE-4D-CT scan was run using protocol C2 (flow rate of 3.0 ml/s; a scan delay of 20 s). Homogeneous enhancement of lesion; the enhancement of mediastinal lymph nodes were obvious. Target volume visibility was classified as positive (Fig. 3).

### Case 3

**Fig. 4:**
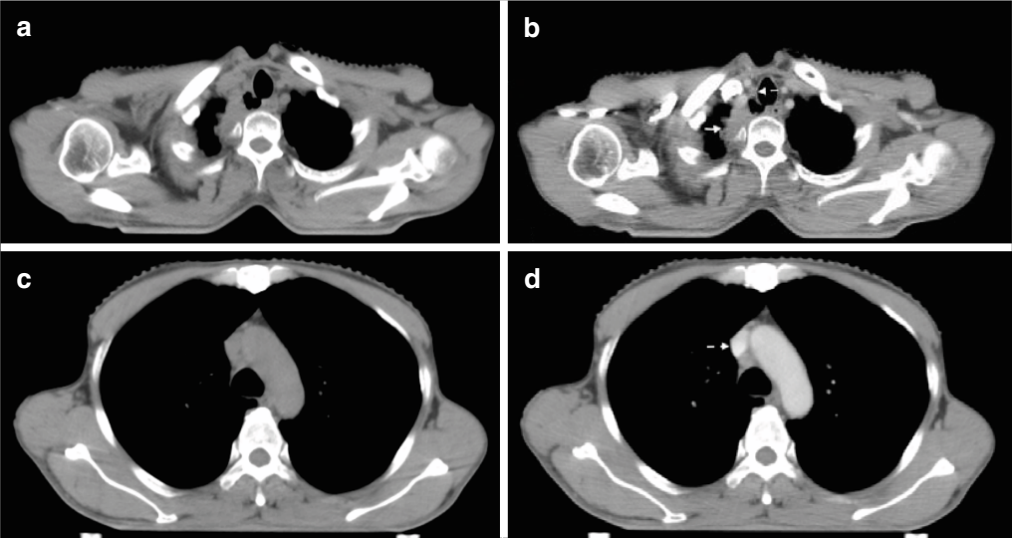
The Hounsfield units (HUs) of lesion in the plain 3D-CT (**a**) and CE-4D-CT (**b**) were 40 ± 6 and 62 ± 13, respectively; and the HUs of upper mediastinal lymph nodes in the plain 3DCT (**c**) and CE-4D-CT (**d**) were 33 ± 11 and 63 ± 16, respectively. The lesion (*solid arrow*) and nodes (*dotted arrow*) were clearly identified

Postoperative metastasis of gingival squamous cell carcinoma in the right upper lung and upper mediastinum. The CE-4D-CT scan was run using protocol C2 (flow rate of 3.0 ml/s; a scan delay of 20 s). The lesions and lymph nodes were significantly enhanced in CE-4D-CT compared with the plain 3D-CT, and the visibility was classified as positive (Fig. 4).

### Case 4

**Fig. 5:**
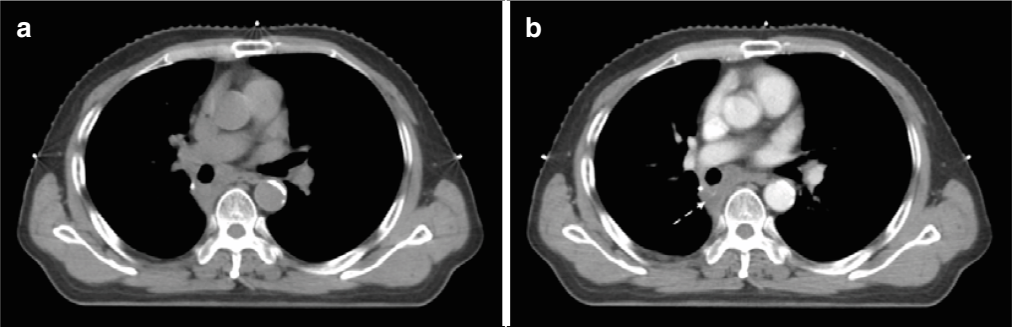
The Hounsfield units (HUs) of hilar lymph nodes in the plain 3D-CT (**a**) and CE-4D-CT (**b**) were 24 ± 18 and 37 ± 19, respectively. Lymph nodes showed low enhancement, but peripheral vessels were highly enhanced, and the lymph nodes (*dotted arrow*) were clearly identified

Postoperative right primary bronchial small cell-squamous cell lung cancer, hilar lymph nodes remain. The CE-4D-CT scan was run using protocol A2 (flow rate of 2.0 ml/s; a delay of 15 s). The hilar lymph nodes showed low enhancement, but its boundary were still identified, the visibility of hilar lymph nodes was classified as positive (Fig. 5).

## Discussion

To minimize the artefacts resulting from respiratory motion, contrast-enhanced 3D-CT diagnostic images are captured while the patients hold their breath, and the most commonly used technique for adjusting the amount of iodine mass based on the body weight is the use of a 1:1 linear scale, which indicates that these values can be optimized for each patient in our institution. However, due to the characteristics of long scanning time of 4D-CT, these contrast-enhancement 3D-CT parameters that directly apply to 4D-CT scans did not work well for the contrast enhancement of vessels. A long scanning duration requires injection of greater doses of iodine compared to a short scanning duration in order to achieve the same magnitude of enhancement. It is theoretically possible to solve this problem by administering patients multiple doses of the contrast agent. Unfortunately, this may not be possible due to the toxicity of excessive amounts of contrast agents. Thus, in this study, we used the diagnostic protocol for acquiring CT images to plan the weight-equivalent contrast agent doses. Because the body weight range and mean weight of our patients were lower than patients in North America and Europe, the volume of the contrast agent used in our study is far less the volumes of 150 ml or 140 ml reported in the literature [13, 15].

In addition to the volume of the contrast agent, other factors such as flow rate of iodine injection, scan delay time, iodine concentration, heart rate, breathing frequency and cardiac output, might also affect the quality of enhancement in 4D-CT scanning [21]. The flow rate of iodine injection and scan delay time were the most important factors which were modified for patients in 4D-CT enhancing scans [22–30]. The administration of changing flow rate and delay time can easily be implemented.

There was no universal agreement in the literature on what HU level is adequate for vessel enhancement. Goodman et al. [31] suggested that the optimally absolute CT value for venous enhancement should be 80 HU or greater. In our study, the enhancements of vessels in the neck and mediastinum are different. If the targets are focused only on the mediastinum, the enhanced values of pulmonary veins, thoracic aorta and pulmonary were more satisfactory in groups A1 and A2, where the HUs of those vessels ranged from 112 to 291, and all grades are greater than 3. Therefore, we suggest that the scanning protocol A1 (flow rate of 2.0 ml/s, a scan delay of 15 s) and A2 (flow rate of 2.0 ml/s, a scan delay of 20 s) provide higher quality contrast-enhancing images for the mediastinal region of interest, as shown in case 1 and 4. Furthermore, when we were interested in the superior mediastinum and supraclavicular region, the HUs of carotid artery and jugular vein ranged from 104 to 353 HU in groups C2 (flow rate of 3.0 ml/s, a scan delay of 20 s) and C3 (flow rate of 3.0 ml/s, a scan delay of 25 s), and all grades of those vessels are greater 3. The protocols C2 and C3 are recommended for the superior mediastinum and supraclavicular regions—as they allowed better identification of tumor lesion, metastatic lymph node and vessels located in this region compared with other protocols, e.g., case 2 and 3.

In our study, the dose of the contrast agent used in this paper did not yield satisfactory enhancement over the entire scan range. The main reason may be that the scanning time is long, but the amount of contrast agent was also too small. If satisfactory enhancement results over the entire scanning range are to be obtained, more scanning parameters may need to be changed, e.g., increasing the volume of the contrast medium, changing the scanning direction.

Some limitations of our study should be mentioned. First, we did not evaluate patient-related factors, such as cardiac output and cardiovascular circulation, which are important as they could affect the duration of contrast enhancement [32]. Second, the scanning protocol for 4D-CT imaging should be individualized for each patient. To achieve the maximum benefits of enhanced 4D-CT, we must be aware that contrast material administration and scan timing protocols need to be optimized by taking into consideration multiple interrelated factors that could affect contrast enhancement and duration.

Although 4D-CT is currently standard in radiotherapy to assess patient-specific respiratory motion, it is not without limitations, e.g., motion artefacts caused by irregular breathing patterns, high radiation dose to the patient due to increased scan time, and poor soft-tissue contrast. Therefore, respiratory-correlated 4D-MRI has gained interest as an alternative to 4D-CT with excellent soft-tissue contrast, no radiation exposure, and relatively insensitivity to target motion [33–40]. Compared to 4D-CT, the potential of 4D-MRI-based contouring will be better at identifying hilar solid tumors, mediastinal lymph nodes, and mediastinal/chest wall invasion, and better consistency in delineating organs at risk (OARs) such as the lungs, esophagus, heart, spinal cord, major vessels, and chest wall. Unfortunately, at present 4D-MRI is still only used in the research setting since no MRI vendors currently offer 4D-MRI products, even though several methods have been proposed especially over the last 3 years [36–40].

In summary, the clinical goal of contrast medium administration is to achieve adequate enhancement, while exposing the patient to the lowest radiation dose possible and administering the lowest amount of iodine. The protocol of contrast medium volume equal to the patient’s weight, flow rate of 2 ml/s, and a scan delay of 15 or 20 s yield an acceptable degree of enhancement for the interest regions of mediastinum and hilum; if we are interested in the region of superior mediastinum and supraclavicular fossa, the protocol of contrast medium volume equal to the patient’s weight, flow rate of 3 ml/s, and the scan delay of 20 or 25 s is recommended.
